# Changes in Attitudes toward Mental Illness in Healthcare Professionals and Students

**DOI:** 10.3390/ijerph16234655

**Published:** 2019-11-22

**Authors:** Yin-Yi Lien, Hui-Shin Lin, Chi-Hsuan Tsai, Yin-Ju Lien, Ting-Ting Wu

**Affiliations:** Department of Health Promotion and Health Education, National Taiwan Normal University, 162, Heping East Road Section 1, Taipei 106, Taiwan

**Keywords:** mental illness, stigma, attitude, healthcare providers

## Abstract

Mental-illness-related stigma not only exists in the public but also in healthcare systems. Healthcare providers (HCPs) who have stigmatizing attitudes or behaviors might be thought of as a key barrier to mental health service use, and influence the quality of healthcare. Although cumulative projects have been conducted to reduce stigma related to mental illness among HCPs around the world, little is known about whether the attitudes of HCPs toward mental illness have changed over time. Research on this topic is mixed with respect to whether attitudes of HCPs toward mental illness have become more or less positive. The aim of the current study was to help clarify this issue using a cross-temporal meta-analysis of scores on the Social Distance Scale (SDS), Opinions about Mental Illness (OMI), and Community Attitudes towards Mental Illness (CAMI) measures among health care professionals and students (*N* = 15,653) from 1966 to 2016. Our results indicated that both social distance (*β* = −0.32, *p* < 0.001) and attitudes (*β* = 0.43, *p* = 0.007) of HCPs toward mental illness have become increasingly positive over time. These findings provide empirical evidence to support that the anti-stigma programs and courses have positive effects on HCPs and can inform future anti-stigma programs focusing on improving the attitudes of HCPs toward mental illness, thereby improving the quality of healthcare provided.

## 1. Introduction

Mental-illness-related stigma is a focus of global public health problems. To challenge stigma associated with mental illness, the World Psychiatric Association (WPA) constructed a global program known as “Open the Doors” to fight the stigma and discrimination of mental illness in 1996 [1]. Many countries have also conducted mental health campaigns. For example, the Australian campaign “Beyond Blue” was established to address depression-related issues and promote awareness among the community [2]. A national campaign called “Time to Change”, which aimed to reduce stigma and discrimination against people with mental health disorders, was launched in 2009 in England [3]. The German campaign “Nuremberg Alliance against Depression” was an intervention program to increase awareness among the public [4]. Time trend studies have evaluated the effects on attitudes toward people with mental illness among the public, and the inconsistent results have been found, with evidence of positive change [5,6,7,8], negative change [8,9,10], or no change [4,8,9,10,11]. The evolution of public attitudes towards people with mental illness has mainly been studied in Western countries (e.g., Germany [4,8,9], Australia [5], England [5,7], and Sweden [6]). Little is known about the change of public attitudes towards mental illness in non-Western countries.

Stigmatizing attitudes are not only confined to the public, but are also prevalent among healthcare providers (HCPs) [12,13]. Accumulating evidence reveals that many people with mental illness report that HCPs, working on both mental and physical health services, are an important source of stigma and discrimination in many countries worldwide [14,15]. Mental-illness-related stigma within the healthcare system and among HCPs has been identified as a major barrier to treatment and recovery as well as a significant source of poorer quality of physical care for persons with mental illness [12,16]. In other words, stigmatizing attitudes or behaviors by HCPs have the potential to lead to a lack of attention to patients’ medical needs, mismanagement of patients with mental illness, and even social marginalization [17].

Furthermore, another major issue regarding stigma toward mental illness among HCPs is that it might cause staff shortages in psychiatry. A systematic review indicated that underlying stigma among medical students towards mental illness has been suggested as an influential factor in shaping the negative views toward a career in psychiatry [18]. Psychiatry has been facing a shortage of specialists [19,20], and the shortage of psychiatrists might cause a growing mental health care system crisis. Research has shown that the shrinking psychiatrist workforce was likely to affect access to care for people with mental illnesses [21]. Under the circumstances, mental-illness-related stigma is increasingly seen as a fundamental cause of population health inequalities and a major challenge for public health.

The problem of mental illness-related stigma within healthcare is an area receiving increased attention and concern [16,17,22]. A great amount of effort has been made around the world to reduce mental-illness-related stigma among HCPs. Education Not Discrimination (END) is one of the components of the Time to Change program, which aims to reduce mental health stigma among healthcare professionals and professional students [23]. Furthermore, an anti-stigma initiative of the Mental Health Commission of Canada (MHCC) known as Opening Minds (OM) has conducted a large series of evaluations of anti-stigma programs targeting various HCPs since 2009 [24]. The OM program has had a dual focus addressing stigma within healthcare as a workplace, as well as addressing stigma within consumer–provider interactions and quality of care. There is substantial research evaluating the attitude toward mental illness among HCPs over the decades in Western [25,26,27] and non-Western countries [28,29]. However, questions remain as to whether these changes in the attitudes of HCPs toward mental illness are moving in a positive direction and whether the changes are influenced by regions. Under the circumstances, there is a need to determine the evolution of the attitudes towards mental illness among HCPs. 

## 2. Method

### 2.1. Search Methods

We conducted a literature search in the electronic databases PubMed, MEDLINE, PsychINFO, PsycARTICLES, and ERIC, using the terms (“stigma” OR “knowledge” OR “stereotype” OR “attitude” OR “prejudge” OR “behavior” OR “discrimination” OR “social distance”) AND (“mental illness” OR “mental disease” OR “mental health” OR “mental health literacy” OR “psychiatry illness” OR “psychiatry disorder” OR “schizophrenia” OR “depress *”) AND (“student” OR “professional” OR “clinicians” OR “physicians” OR “health staff” OR “medical personnel” OR “healthcare provider”) AND (“survey” OR “scale” OR “measurement”). We searched for peer-reviewed journal articles regarding attitudes toward mental illness in a diverse group of healthcare professionals and students that appeared until 28 February 2019. In addition, we also performed a manual search of references cited by the published original studies, relevant reviews, and meta-analysis articles. Furthermore, we contacted the experts in the field of attitude research and asked them about any relevant studies to expand the initial search. Before data collection, ethical approval was obtained from the Institutional Review Board of National Taiwan Normal University (ID: 201808HS12).

### 2.2. Study Selection

**Figure 1:**
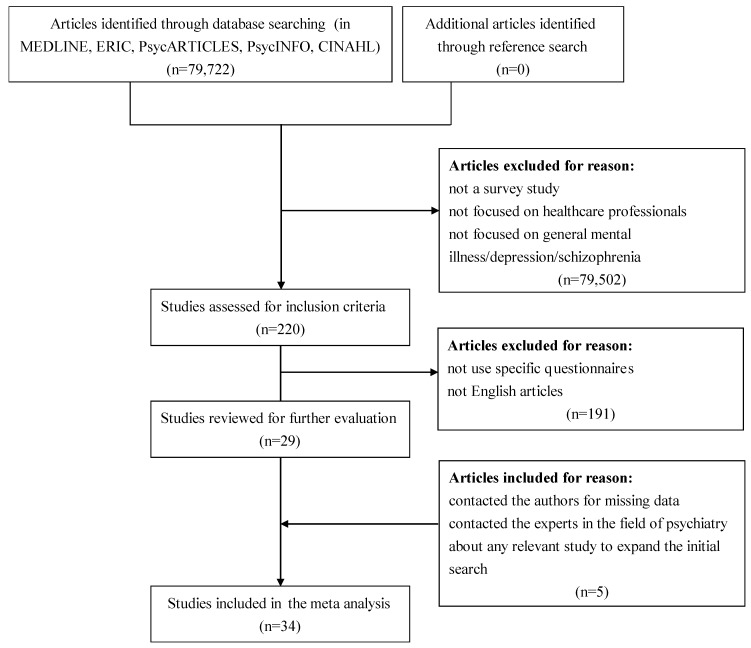
Flowchart of study selection.

This review followed PRISMA guidelines [30], and the protocol is registered with the PROSPERO database of systematic reviews (PROSPERO: CRD42018112875) [31]. Figure. 1 describes the flow of candidate and eligible articles. We retained all reports on studies that met the following criteria. First, the focus of the study was on the healthcare professionals and students. Studies investigating the beliefs or attitudes of professionals or students in healthcare fields (e.g., medical, nursing, social work, psychology, pharmacy, occupational therapy, physical therapy) were included. Second, there are many measures to assess the attitudes and beliefs among healthcare professionals and students, such as the Social Distance Scale (SDS) [32], Reported and Intended Behaviour Scale (RIBS) [33], Opinions about Mental Illness (OMI) [34], Community Attitudes towards Mental Illness (CAMI) [35], Mental Illness Clinicians Attitude (MICA) [36], and Opening Minds Stigma Scales for Health Care Providers (OMS-HC) [37]. Typically, the Social Distance Scale is regarded as a proxy measure of mental-health-related stigma [32,38,39,40,41]. OMI, CAMI, MICA and OMS-HC were designed to assess attitudes towards people with mental illness. Especially, MICA and OMS-HC were recently developed for healthcare professionals. As we were interested in sustained time trends of the attitudes and beliefs of healthcare professionals and students, MICA and OMS-HC were not included in this study because the two measures have not been in use for long enough. Accordingly, we included studies in which the outcome was measured using the Social Distance Scale (SDS), Opinions about Mental Illness (OMI), and Community Attitudes towards Mental Illness (CAMI). Third, we included only survey studies for the following reasons: (a) For our study period we found only two intervention studies used SDS, OMI or CAMI as outcome measures. The publications are scarce in this period. (b) In survey studies the samples are usually comprised of a wide range of study participants, but in intervention studies small numbers of people are tested. (c) Additionally, survey studies have been conducted all over the world, whereas intervention studies were conducted in only a few countries. Fourth, we only included studies published in English. We also attempted to contact authors for missing data and the experts in the field of psychiatry about any relevant studies to expand the initial search. The contents of abstracts or full-text manuscripts identified through the literature search were reviewed independently by two authors in duplicate to determine whether they met the eligibility criteria for inclusion. Disagreements between two authors were resolved by consensus with discussion.

### 2.3. Relevant Measures

There are a number of measures assessing attitudes and social distance toward mental illness [42,43]. With regard to investigating attitudes toward mental illness, one popular measure is the Opinions about Mental Illness (OMI) [34]. The 51-item OMI assesses five domains of attitudes toward people with mental illness, including (1) authoritarianism; (2) social restrictiveness; (3) benevolence; (4) mental hygiene ideology; and (5) interpersonal etiology. Participants are asked to rate each item on a 6-point Likert scale ranging from strongly agree to strongly disagree. A modified version of the OMI scale called the Community Attitudes towards Mental Illness (CAMI) scale [35] has three out of four factors in common with the OMI scale: authoritarianism, benevolence, and social restrictiveness. CAMI can detect the attitude of accepting psychiatric patients in a community. It requires the participants to answer items on a 5-point Likert scale ranging from strongly agree to strongly disagree. A high score on the subscales and the total scale indicates positive attitudes toward mental illness.

The Social Distance Scale (SDS) [32] is commonly used to measure social distance toward mental illness, which is assessed as the level of desired future contact with people with mental health problems. Many studies have established that the SDS has validity and reliability [32,44,45]. The original SDS contains seven items, and each item asks the participant to use a 4-point Likert scale (1 = “Definitely Unwilling” to 4 = “Definitely Willing”). As such, possible scores range from 7 to 28, with higher scores indicating more social distance [32].

### 2.4. Cross-Temporal Meta-Analysis

Cross-temporal meta-analysis [46,47,48,49,50] is similar to traditional meta-analysis in the procedures for identifying and collecting data for studies. Instead of computing an effect size for each study as in traditional meta-analysis, cross-temporal meta-analysis records means, standard deviations, data collection year of study (as two years prior to publication, unless the year was otherwise noted in the article), as used by previous studies [51,52], and other study characteristics are also coded (e.g., sample number, region). In our cross-temporal meta-analysis we were interested in the relationship between the mean scores on the outcome measures and the year that these data were collected. In addition, we weighted the data in two ways. First, studies with larger sample sizes providing better estimates of the population mean had a stronger influence on our findings [53]. Second, studies with smaller variances had a stronger influence on the final results. Cross-temporal meta-analysis ultimately provides an index of the degree to which scores on a measure of outcome have changed over time.

We analyzed how attitudes and social distance scores have changed over time, primarily by examining correlations between mean scores and year of data collection. Subgroup analysis was used to examine whether the observed effects was different by regions. Cross-temporal meta-analyses were performed using Comprehensive Meta-Analysis Version 3 (Biostat, Englewood, NJ, USA) [54]. We fit random-effects models, which took into account the between-study variations, to study the factors that might affect social distance and attitude. Because of the different scales and measurement items in the studies, we adjusted scores before conducting analyses [9]. This technique was used to compute the correlation between attitudes and social distance mean scores and year.

## 3. Results

### 3.1. Data Identification and Extraction

Applying our study criteria, we identified 34 studies that used SDS, OMI, or CAMI and met the inclusion criteria (see Figure 1): 18 studies using the SDS, 6 studies using OMI, and 10 studies using CAMI. These studies were based on a combined sample size of 15,653 participants. Characteristics of each study included in the review are documented in Table 1 and Table 2.

### 3.2. Correlation between Mean Scores of Social Distances, Attitude, and Years

**Figure 2:**
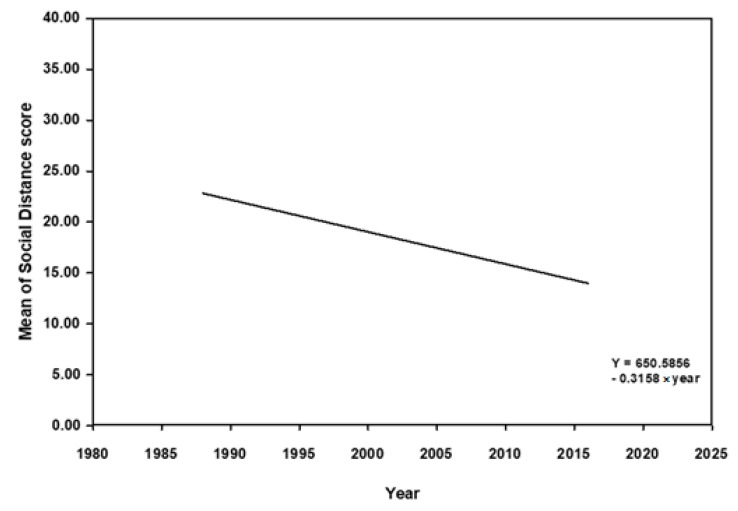
Meta regression of mean of Social Distance Scale score and year of data collection from 1988 to 2016.

**Figure 3:**
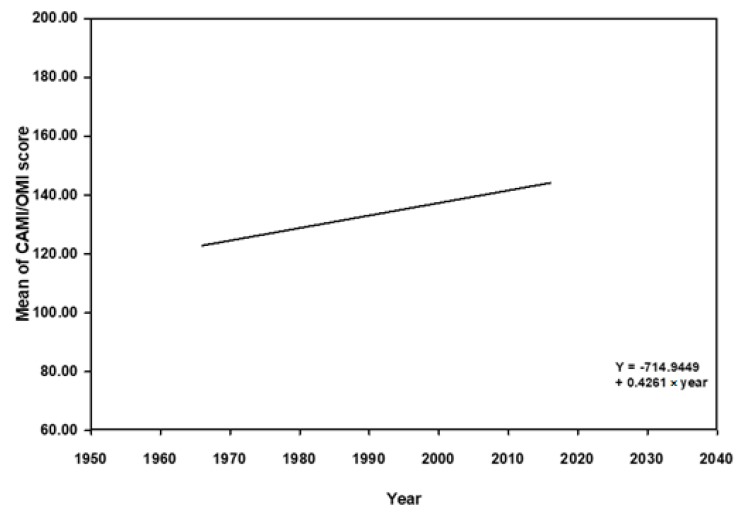
Meta regression of mean of CAMI/OMI score and year of data collection from 1966 to 2016. CAMI: Community Attitudes towards Mental Illness scale; OMI: Opinions about Mental Illness scale.

The cross-temporal meta-analysis showed that the mean scores of social distance and attitudes were positively associated with the year of data collection (*β* = −0.32, *p* < 0.001; *β* = 0.43, *p* = 0.007), indicating that the desire of social distance and the attitudes toward people with mental illness in healthcare professionals and students become positive over the period 1966–2016 (Figure 2 and Figure 3). To further examine the magnitude of change in SDS and attitude scores, we calculated the size of increase in scores over time using the regression equation weighted by *w*. The regression equation used the algebraic formula *Yx*_1_ = *C*_1_ + *Bx*_1_, where *Yx*_1_ is the average SDS score for a particular year of interest, *x*_1_ is the year of interest, *B* is the beta coefficient of −0.32 (*p* < 0.001), and *C*_1_ is the equation constant of 650.59. We used the *r^2^* estimator to conclude that only 17% of the variance in effects was explained by the model. The regression equation of attitude was *Yx*_2_ = *C*_2_ + *Bx*_2_, where *Yx*_2_ is the average attitude score for a particular year of interest, the beta coefficient was 0.43 (*p* < 0.05), and *C*_2_ is the equation constant (−714.94). *r^2^* was 0.16, which means 16% of the variance in effects was explained by this model.

Considering regional difference, additional subgroup analysis was conducted only for social distance and not for OMI and CAMI because there was only one study using CAMI as an outcome measure in the non-Western country (i.e., Nigeria). The subgroup analysis indicated that greater mean scores of social distance were also associated with the later years of data collection in both the Western countries (*n* = 14; *β* = −0.27, *p* = 0.001) and non-Western countries (*n* = 5; *β* = −0.73, *p* = 0.048). This reveals that the desire of social distance from people with mental illness in healthcare professionals and students became more favorable with the passage of time, regardless of region. Compared with the findings from the original cross-temporal meta-regression model, similar findings were found after including region as a covariate. Our findings suggest that the correlation between the year of data collection and social distance was also independent of region.

## 4. Discussion

Summarizing our findings, this study indicates that over a half century, HCPs’ attitudes toward mental illness have increased considerably. Moreover, there has been a significant reduction of social distance from people with mental illness among HCPs over the past three decades. Meanwhile, our study showed that in both Western and non-Western countries, the attitudes among HCPs toward mental illness have improved in recent decades. Reducing mental-illness-related stigma among HCPs has become a global campaign [13]. The deleterious impacts of stigma in healthcare have promoted increased calls to action for health organizations to take leadership roles in tackling the problem [17,84], such as the OM initiative and Time to Change programs in Western countries. Of note, there were also some effective interventions which aimed to reduce the mental-illness-related stigma among HCPs in non-Western countries such as Hong Kong [28], Japan [29,85], South Africa [86], and Turkey [87,88,89]. In addition, compared with traditional education, the involvement of consumers in the education (i.e., contacting people with mental illness) of HCPs has been identified as a potentially effective strategy in influencing more positive attitudes toward consumer involvement in mental health services [14,90]. Furthermore, an educational strategy called problem-based learning (PBL) is a common newer teaching technique used in medical education in recent years. Research has suggested that the PBL method has played an effective role in the development of positive attitudes toward psychiatric nursing and patients as well as in the acquisition of the basic skills of psychiatric nursing [91]. Under the circumstances, participating in anti-stigma programs and modernizing medical education might help HCPs generate positive attitudes toward mental illness.

Although we found these positive improvements, the results of our study have some limitations. First, mental illness is a general term for a group of illnesses that may influence a person’s thoughts, perceptions, feelings, and behaviors. We only included studies evaluating stigma toward mental illness, schizophrenia, and depression in our criteria since these are most common diagnoses used in the mental health campaigns to reduce mental-illness-related stigma among HCPs. A range of diagnosis-based specific mental disorder conditions (e.g., bipolar disorder and alcohol use disorder) could be a target for future research. Second, potentially relevant studies were not included in this study due to lack of access to an English version. However, most studies on evaluating HCPs’ stigma toward mental illness took place in Western countries. The findings might not be easily translated to Eastern cultures. Third, as in any meta-analysis, interpretations of the results of this study are limited to the data reported by authors. Specifically, many authors do not report the specific year of data collection, the ethnicity of their participants, or the means and standard deviations for all variables. However, the goal of this cross-temporal meta-analysis was to examine the relationship between time and attitude. This study also could not determine whether the change in attitude was a purely generational effect or a time-period effect. As with any time-lag study including people of only one age group, we cannot know if those in other age groups also changed. Finally, the use of an attitude scale to assess outcome might be influenced by socially desirable responding.

## 5. Conclusions

This study provides further evidence in support of the importance of global and national programs and new medical education methods in eliminating stigma toward mental illness among HCPs. The findings also suggest that these efforts improve positive attitudes toward mental illness and reduce the social distance from mentally ill people. As actions to fight the stigma toward mental illness have continued, new trend analyses tracking present and future attitude changes are necessary. Future research might focus on monitoring and evaluating the trends nationally as well as globally and determining if there are differences in cultural needs, reception, and reactions to different campaign messages.

## Figures and Tables

**Table 1:** Summary of characteristics of the 18 Social Distance Scale (SDS) studies.

Study Author, Publication Date	Country	Year of Data Collection	Group	*N*	Total Score, *μ* (SD)
Crismon, 1990 [25]	United States	1988	Pharmacists	165	15.87 (4.08)
Bell et al., 2006 [55]	Australia	2004	Third-year pharmacy students	216	18.75 (5.04)
			Pharmacy graduates	232	18.52 (5.00)
Volmer et al., 2008 [26]	Estonia	2006	Pharmacy students	157	20.36 (3.88)
Bell et al., 2010 [56]	Australia, Belgium, India, Finland, Estonia, Latvia	2006	Pharmacy students in India	106	18.75 (3.57)
			Pharmacy students in Australia	241	19.65 (3.97)
			Pharmacy students in Finland	130	18.05 (3.12)
			Pharmacy students in Estonia and Latvia	70	20.90 (4.04)
			Pharmacy students in Belgium	102	19.61 (2.92)
Hanzawa et al., 2012 [57]	Japan	2009	Psychiatric nurses	215	19.76 (4.30)
Loch et al., 2013 [58]	Brazil	2009	Psychiatrists	1414	14.00 (3.58)
Mittal et al., 2014 [59]	United States	2011	Mental health providers	205	14.87 (6.01)
			Primary care providers	146	16.23 (6.89)
Reavley et al., 2014 [60]	Australia	2012	General Practitioners	518	14.14 (5.18)
			Psychiatrists	506	14.14 (5.67)
			Psychologists	498	12.25 (4.48)
Amarasuriya et al., 2015 [61]	Sri Lanka	2013	Medical students	605	13.03 (4.02)
Dabby et al., 2015 [62]	Canada	2012	Psychiatrists	68	10.47 (3.36)
Mak et al., 2015 [28]	Hong Kong	2011	Nursing professionals ^a^	209	16.31 (5.06)
			Social work professionals ^a^	150	13.23 (4.29)
			Medical professionals ^a^	149	16.87 (5.13)
			Nursing students ^a^	203	12.81 (4.99)
			Social work students ^a^	207	13.86 (5.04)
			Medical students ^a^	60	13.30 (4.88)
			Nursing professionals ^b^	186	18.55 (4.77)
			Social work professionals ^b^	154	15.61 (4.34)
			Medical professionals ^b^	201	19.74 (4.96)
			Nursing students ^b^	203	16.17 (4.99)
			Social work students ^b^	185	17.99 (5.71)
			Medical students ^b^	52	16.73 (5.55)
O’Reilly et al., 2015 [63]	Australia	2009	Pharmacists	186	17.81 (3.79)
Chiba et al., 2016 [29]	Japan	2012	Psychiatrists, nurses, clinical psychologists, pharmacists, occupational therapists, social workers	307	15.22 (4.75)
Smith et al., 2017 [64]	United States	2012	Primary care nurses	91	15.83 (4.67)
			Primary care physicians	55	16.88 (4.05)
			Mental health nurses	67	15.01 (4.81)
			Psychiatrists	62	15.92 (5.07)
			Psychologists	76	13.89 (3.91)
Pranckeviciene et al., 2018 [65]	Lithuanian	2015	Students (social work)	296	18.14 (3.76)
			Students (psychology)	419	17.18 (3.64)
			Social workers	111	17.43 (4.00)
			Psychologists	122	16.61 (3.37)
Tay et al., 2018 [27]	United Kingdom	2015	Psychologists	678	12.18 (3.71)
Tillman et al., 2018 [66]	United States	2016	Students (social work)	104	11.90 (3.77)
			Students (counseling)	87	11.04 (3.20)
			Students (psychology)	111	11.90 (3.94)
			Social workers	23	10.01 (3.59)
			Counselors	34	11.02 (3.24)
			Psychologists	38	12.13 (3.16)
Perlman et al., 2019 [67]	Australia	2016	Nurses	168	15.82 (3.76)

^a^ The outcome measure is social distance of depression; ^b^ The outcome measure is social distance of schizophrenia.

**Table 2:** Summary of characteristics of the 16 OMI/CAMI Studies.

Study Author, Publication Date	Scale	Country	Year of Data Collection	Group	*N*	Total Score, *μ* (SD)
LeMay et al., 1968 [68]	OMI	United States	1966	Counselor candidates (male)	31	134.50 (14.66)
				Counselor candidates (female)	50	134.29 (13.13)
Levine et al., 1972 [69]	OMI	Great Britain, Czechoslovakia, Germany	1968	Physicians (British)	181	127.23 (19.52)
				Physicians (Czechoslovakian)	103	110.75 (18.52)
				Nurses (West German)	80	120.69 (22.52)
				Nurses (British)	188	128.30 (20.18)
				Nurses (Czechoslovakian)	116	105.35 (19.31)
Kirkby et al., 1979 [70]	OMI	Australia	1977	Medical practitioners	37	129.18 (20.25)
Murray et al., 1999 [71]	OMI	United States	1997	Supportive case managers	24	147.48 (16.56)
				Intense case managers	23	135.87 (17.30)
Smith et al., 2008 [72]	CAMI	United States	2006	Health professionals and medical students	168	113.87 (20.83)
Arvaniti et al., 2009 [73]	OMI	Greece	2006	Health professionals and medical students	580	147.38 (25.85)
Smith et al., 2010 [74]	CAMI	United States	2008	Mental health students	58	143.10 (15.59)
				Mental health professionals	58	141.40 (17.19)
Chambers et al., 2010 [75]	CAMI	Finland, Lithuania, Ireland, Italy, Portugal	2007	Nurses	810	134.00 (20.74)
Guise et al., 2010 [76]	CAMI	United Kingdom	2009	Nurses	81	135.50 (17.07)
O’ Connor et al., 2013 [77]	CAMI	Ireland	2010	Medical students (third year)	140	159.20 (14.60)
				Medical students (final year)	145	158.50 (16.50)
Kopera et al., 2015 [78]	OMI	Poland	2011	Psychiatrists, psychotherapists Medical students	57	147.80 (13.96)
Winkler et al., 2016 [79]	CAMI	Czech Republic	2014	Medical doctors	1200	142.22 (16.30)
Janouskova et al., 2017 [80]	CAMI	Czech Republic	2016	Medical students	457	163.56 (18.68)
Mosaku et al., 2017 [81]	CAMI	Nigeria	2013	Health workers	112	115.60 (19.96)
Siqueira et al., 2017 [82]	CAMI	Brazil	2014	Health professionals	246	113.20 (14.80)
Cremonini et al., 2018 [83]	CAMI	Italy	2016	Health care professionals	120	160.77 (15.60)

OMI: Opinions about Mental Illness scale; CAMI: Community Attitudes towards Mental Illness scale.
